# The Mission to Lepers in India and the East

**Published:** 1908-02-08

**Authors:** J. A. Bourdillon, Algernon Coote, C. A. Elliott, W. H. Findlay, H. E. Fox, R. Wardlaw Thompson, A. Wingate

**Affiliations:** Sec. W.M.M.S.; Hon. Sec. C.M.S.; D. D., Foreign Sec. L.M.S.; K.C.I.E.


### EDITOR'S LETTER-BOX.

### THE MISSION TO LEPERS IN INDIA AND THE EAST.

Sir,—The present scarcity in India is already severely, and especially, affecting one class of the population, and is causing a serious drain on the resources of the society that exists for their relief. We refer to the homeless and destitute lepers and to the Mission to Lepers in India and the East, of which we are Vice-Presidents, and with whose beneficent work we are well acquainted. The Mission is now responsible for the maintenance of fifty asylums for lepers and twenty homes for their untainted children, and in these some 4,000 sufferers, who would otherwise be a danger and an offence to the community, are being sheltered, fed, and medically relieved, while the children are being educated for lives of usefulness. The society is unquestionably doing a work which is of great national importance, and which is warmly appreciated by the Governments in India.

Of all sections of the community the lepers are the first to feel the pinch of scarcity. The scanty gifts of grain that keep life in them in ordinary times are withheld directly famine threatens. Always on the very verge of actual privation, this forms the turning-point, and to-day there is literally famine amongst the lepers.

The superintendent of the society's largest asylum—at Purula, Bengal, where more than 600 lepers are segregated and supported—wrote in December last that famine prices were already prevailing, and that by the present date rice would be selling at only 6 to 7 seers for the rupee, as compared with 16 to 20 in normal times.

Again, from Chandkuri, in the Central Provinces, we are informed that many more lepers will certainly flock to the asylum as food becomes scarcer, and will swell the numbers beyond the 407 reported a year ago. Similar conditions prevail at many other stations. Owing to the rapid expansion of the society's work in recent years, its funds are sorely taxed even under ordinary conditions; but just now, owing to so large a proportion of its expenditure being for food, it is in urgent need of an increased income, and we earnestly commend it to the practical sympathy of your readers.

Contributions should be addressed to Mr. John Jackson, F.R.G.S., Organising Secretary, 33 Henrietta Street, Covent Garden, London, W.C., and cheques crossed Barclay and Co. We remain, yours faithfully,

J. A. Bourdillon, K.C.S.I.
Algernon Coote, Bart., H.M.L.
C. A. Elliott, K.C.S.I., LL.D.
W. H. Findlay, Sec. W.M.M.S.
H. E. Fox, Hon. Sec. C.M.S.
R. Wardlaw Thompson, D.D., Foreign Sec. L.M.S.
A. Wingate, K.C.I.E.

THE HOSPITAL February 8, 1908.

